# Ultrasound guided continuous Quadratus Lumborum block hastened recovery in patients undergoing open liver resection: a randomized controlled, open-label trial

**DOI:** 10.1186/s12871-019-0692-z

**Published:** 2019-02-18

**Authors:** Qiang Zhu, Li Li, Zhaoyun Yang, Jinmei Shen, Rong Zhu, Yu Wen, Wenwu Cai, Lei Liu

**Affiliations:** 1 Department of Anesthesiology, The Second Xiangya Hospital of Central South University, 139 Renmin Middle Road, Hunan, China; 2 Department of Hepatobiliary Surgery, The Second Xiangya Hospital of Central South University, 139 Renmin Middle Road, Hunan, China

**Keywords:** Liver resection, Continuous quadratus lumborum block, Ultrasound guidance, Postoperative analgesia

## Abstract

**Background:**

Quadratus lumborum (QL) block is increasingly being used as a new abdominal nerve block technique. In some studies of mid and lower abdominal and hip analgesia, continuous QL block achieved favorable outcomes as an alternative to continuous intravenous analgesia with opioids. However, the use of continuous QL block for upper abdominal pain is less well characterized. This study aimed to investigate the effects of continuous anterior QL block (CQLB) on postoperative pain and recovery in patients undergoing open liver resection.

**Methods:**

Sixty-three patients underwent elective open liver resection were randomly divided into continuous anterior QL block (CQLB, *n* = 32) group and patient-controlled intravenous analgesia (PCIA, *n* = 31) group. Patients in CQLB group underwent ultrasound-guided anterior QL block at the second lumbar vertebral transverse processes before general anesthesia, followed by postoperative CQLB analgesia. Patients in PCIA group underwent continuous intravenous analgesia postoperatively. Postoperative numerical rating scale (NRS) pain scores upon coughing and at rest, self-administered analgesic counts, rate of rescue analgesic use, time to first out-of-bed activity and anal flatus after surgery, and incidences of analgesic-related adverse effects were recorded.

**Results:**

Postoperative NRS pain scores on coughing in CQLB group at different time points and NRS pain score at rest 48 h after surgery were significantly lower than those in PCIA group (*P* < 0.05). Time to first out-of-bed activity and anal flatus after surgery in CQLB group were significantly earlier than those in PCIA group (*P* < 0.05). No significant differences of postoperative self-administered analgesic counts, rate of postoperative rescue analgesic usage, or incidences of analgesic-related adverse effects were found between the two groups (*P* > 0.05).

**Conclusions:**

Ultrasound-guided anterior QL block significantly alleviated the pain during coughing after surgery, shortened the time to first out-of-bed activity and anal flatus, promoting postoperative recovery of the patients undergoing open liver resection.

**Trial registration:**

This study has been registered in April 1, 2018 on Chinese Clinical Trail Registry, the registration number is ChiCTR1800015454.

## Background

Liver resection is the main treatment for hepatic space-occupying lesions. Although laparoscopic liver resection has been used in clinical practice, it is still in the developing stage, and open surgery is a more commonly used surgical method [1, 2]. An open liver resection requires a long incision which is traumatic, it is often associated with severe postoperative pain, mainly due to transection and traction on multiple spinal nerves [3, 4]. Postoperative pain causes not only psychological trauma but also increases the incidence of perioperative complications. An ideal analgesic strategy should be effective in alleviating pain, mitigating stress response, have few adverse effects, and facilitating patient recovery. High level epidural analgesia and intravenous opioid analgesia are two common methods used for upper abdominal pain [5]. However, high level epidural block analgesia is technically challenging,and application of opioids may result in many adverse reactions, such as excessive sedation, respiratory depression, and deceleration of gastrointestinal motility [6–8], which are not conducive to rapid recovery. Those disadvantages lead a search for other analgesic strategies.

Quadratus lumborum block is an emerging technique for peripheral nerve blockade, which generates an analgesic effect by unilaterally blocking spinal nerves from T6–T9 to L1-L3 [9], Considering its wide block range, It has been increasingly used for postoperative analgesia in patients undergoing middle and lower abdominal and hip surgery [10–12], and showed satisfactory results no matter in single injection mode or continuous infusion mode. However, application of continuous QL block for upper abdominal pain is less well characterized. Thus, this study aims to evaluate the effect of ultrasound-guided continuous anterior QL block on perioperative pain and postoperative recovery in patients undergoing open liver resection.

## Methods

### Patients

**Fig. 1:**
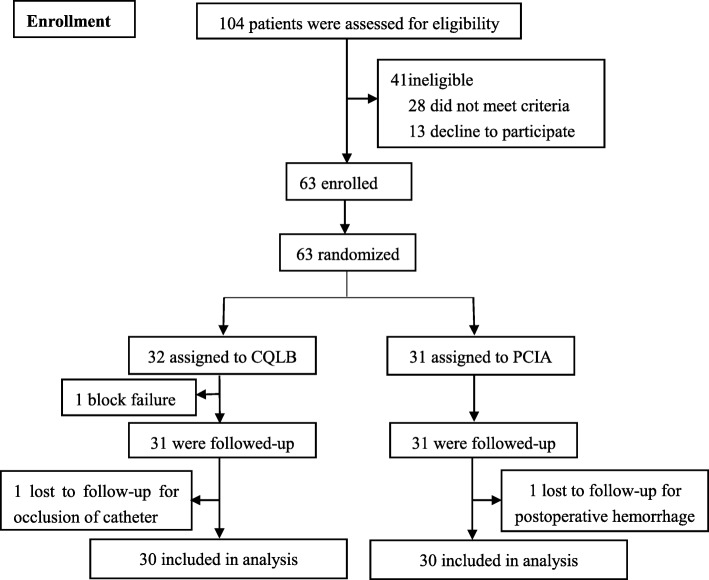
Flow chart of this study. A total of 63 patients were enrolled in this study. One patient from the PCIA group was lost to follow-up due to postoperative hemorrhage, one block failure and one catheter occlusion in the CQLB group were lost to follow-up. Therefore, 30 patients in each group had completed the study

In this randomized controlled trial, patients undergoing elective open liver surgery for liver space-occupying lesions in the Second Xiangya Hospital of Central South University, Changsha, China, from April 1, 2018 to September 30, 2018, were recruited. All patients who meet the criteria and voluntarily signed the written informed consent were randomly allocated to CQLB group (*n* = 32) and PCIA group (*n* = 31) using computer-generated randomization method. A simple randomization schedule was performed and randomization number was generated using Excel 2016 (Microsoft Inc., Redmond, WA). The patients were randomly allocated into 2 groups by the computer-generated randomization schedule (Fig. 1). Because of the invasive nature of the interventions, neither the trial participants nor the investigators were masked to group allocation. The implementation of CQLB, intraoperative anesthesia management and postoperative follow-up were accomplished by three different anesthesiologists, and mutual help were not allowed. CQLB procedures were completed by one experienced anesthesiologist. The research protocol was approved by the Medical Ethics Committee of the Second Xiangya Hospital of Central South University, and has been registered in Chinese Clinical Trial Registry.

Inclusion criteria: Patients (1) Physical status class I–III based on the ASA classification; (2) aged 30–70 years old; (3) with a primary diagnosis of liver space occupying lesions or intrahepatic stones in the intrahepatic bile duct; and (4) use subcostal surgical incision. Exclusion criteria: Patients (1) with severe organ dysfunction (i.e., liver function: child-Pugh score above class B; cardiac function above class II according to the NYHA Classification); (2) coagulation disorders; (3) history of mental illness or drug abuse; (4) clear spinal deformity or anticipated ultrasound anatomical abnormalities; (5) history of infection or trauma at the puncture site; (6) allergy to local anesthetics, and(7)patients in the CQLB group with a block plane below the T7 level at the right abdomen wall 30 min after the CQLB completion.

### Procedures

**Fig. 2:**
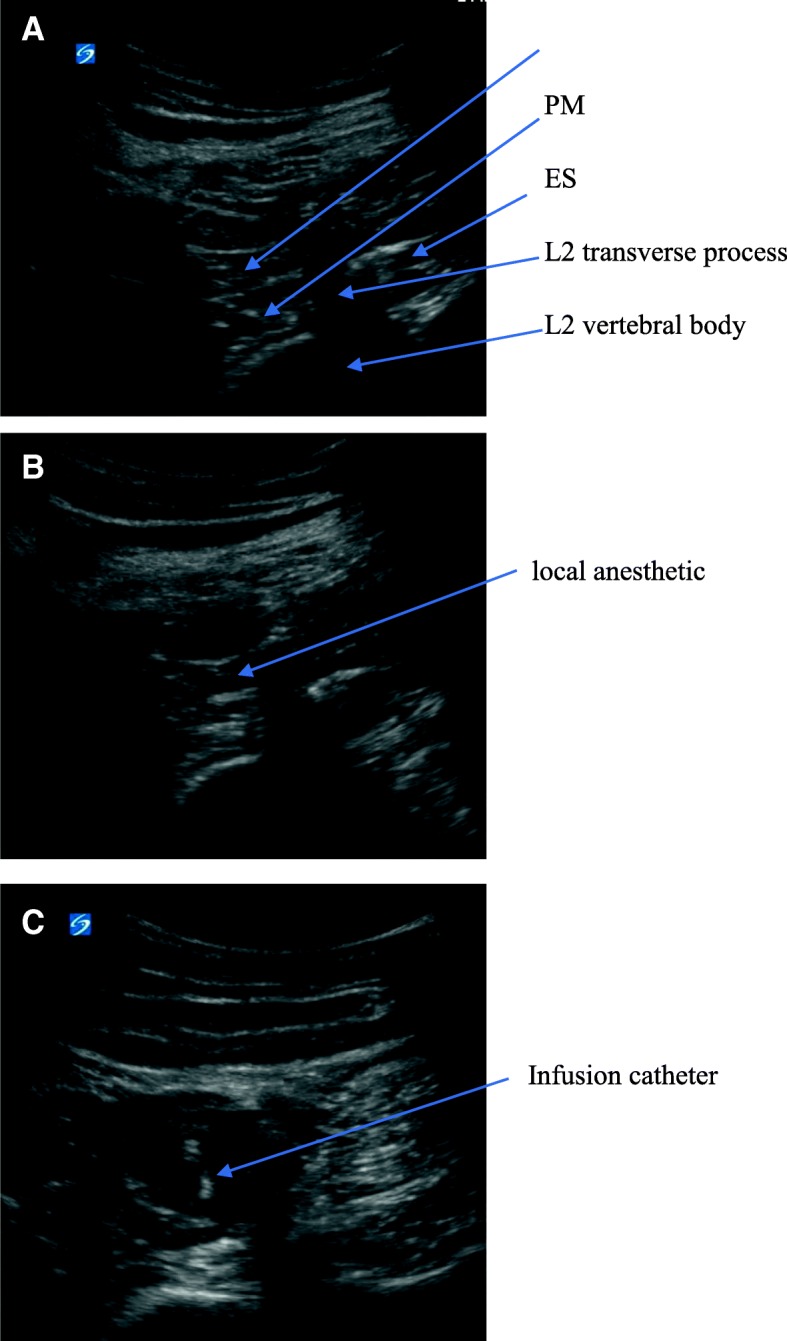
The ultrasound view of the CQLB. QL: Quadratus Lumborum PM: psoas major ES: erector spinae. **a**: ultrasound anatomical structure **b**: spread of local anesthetic. **c**: image of catheterization

After patients entering the anesthesia preparation room, 5-lead electrocardiogram, non-invasive blood pressure and pulse oximetry were monitored, followed by insertion of an Intravenous Catheter for infusion. To ensure safety and efficiency, CQLB was done before anesthesia induction and block range was tested 30 min later. Patients in CQLB group underwent ultrasound-guided anterior QL block at the level of second lumbar vertebra (L2) transverse process as described by Mette Dam MD [13]. Patient was placed in the left lateral position, a low frequency convex probe (SonoSite X-Porte transducer, 2–5 MHz) was placed at the L2 transverse level near posterior axillary line to locate the “sham-rock sign” (Fig. 2a) [13] and fine-tuned to visualize the clearest dividing line between the quadratus lumborum and psoas major. A 18G trocar needle was applied (Contiplex® D model of continuous nerve block Kit, B. Braun Medical Inc.) to puncture using in-plane insertion method from the dorsal to ventral direction. When the tip of the needle just reached the front edge of the quadratus lumborum, approximately 5 ml of normal saline was injected to separate the muscle and visualize it as “separating” on ultrasound view, indicating that the needle tip reached the correct position. Ropivacaine 0.4% at 0.6 ml·kg^− 1^ was injected (Fig. 2b), then, a catheter was placed through the sheath tube at a depth of approximately 5 cm (Fig. 2c). An approximately 3 cm subcutaneous tunnel was made to prevent catheter from dislodging. Patients whose block plane of right abdominal wall above T7 level 30 min after completed the block were considered to have achieved effective blockade and could subsequently undergo anesthesia induction.

After entering the operation room, invasive blood pressure of right radial artery and bispectral index of EEG(BIS) monitor were added before induction. Patients in both groups underwent intravenous induction using 0.06 mg·kg^− 1^midazolam, 0.5 μg·kg^− 1^sufentanil, 0.2 mg·kg^− 1^ etomidate, and 0.1 mg·kg^− 1^vecuronium. The anesthesia was maintained by total intravenous anesthesia approach. Target-controlled infusion of propofol was started immediately after anesthesia induction to maintain the BIS between 40 and 55. Cisatracurium was injected discontinuously to maintain muscle relaxation. Another 0.1 μg·kg^− 1^sufentanil was added 3 min before skin incision, followed by starting target-controlled infusion of remifentanil at an initial concentration of 2 ng·ml^−1^ and was adjusted (8 ng·ml^−1^ maximum) to maintain blood pressure and heart rate (HR) fluctuation within 20% of the baseline value intraoperatively after opening the peritoneum. Transfusion of blood was guided by ASA’s guideline for perioperative blood transfusion and adjuvant therapies [14]. Intravenous injection of 100 mg flurbiprofen and 0.1 μg·kg^− 1^sufentanil were administrated when start to close the abdominal cavity. Once the muscle layer was fully sutured, infusion of remifentanil was stopped and infusion of propofol was reduced to maintain the BIS between 55 and 65 and was terminated at the end of the operation. Analgesics used in PCIA group were 2.5 μg·kg^− 1^sufentanil and 8 mg ondansetron diluted to a final volume of 100 ml in normal saline. Parameters of the analgesic pump included 2 ml·h^− 1^ continuous infusion volume (0.05 μg·kg^− 1^·h^− 1^), 2 ml bolus dose, 10 min lockout time, and 8 ml·h^− 1^maximum infusion volume. Analgesic used in CQLB group was 250 ml 0.2% ropivacaine, and the parameters of the analgesic pump were 5 ml·h^− 1^ continuous infusion volume, 5 ml bolus dose, 15 min lockout time, and 20 ml·h^−1^ maximum infusion volume. If postoperative NRS pain score at rest was greater than 4 and showed no relieve after 2 bolus doses, Tramadol 100 mg was injected as a rescue method.

SBP, DBP and HR of the two groups of patients were monitored after settling in the preparation room (T0), 5 min before anesthesia incision (T1), before opening the parietal peritoneum (T2), 0.5 h after recovery from anesthesia (T3), and at postoperative 2 h(T4), 6 h(T5), 12 h(T6), 24 h(T7), and 48 h(T8). The duration of operation, intraoperative propofol and remifentanil consumption, and the time of recovery from anesthesia were recorded. In addition, the postoperative NRS pain score on coughing and at rest and Ramsay sedation scale at each time point, postoperative self-administered analgesic counts, rate of rescue analgesic usage, time to first out-of-bed activity after surgery, time to first flatus, and the incidence of analgesic-related adverse effects, such as postoperative nausea, vomiting, and respiratory depression (RR < 10, PaO_2_, <94% during O_2_ inhalation), were recorded.

### Statistical analysis

Pilot study of 20 patients in each group showed that the time for first anal flatus in CQLB group was 60.5 ± 20.8, whereas it was 75.2 ± 14.3 in the PCIA group. Giving α = 0.05 (statistically significant level), 1-β = 0.8(power of a test), a sample size of 25 patients in each group was needed calculated by Gpower 3.1(Heinrich-Heine-University, Düsseldorf, German). To compensate for potential exclusion or withdrawals, we recruited 63 patients in total. GraphPad prism 7.0 (GraphPad Software, San Diego, CA, U.S.) was used for statistical analysis. Normally distributed data are presented as mean ± standard deviation, and the comparison between groups were performed using independent sample t-test and the comparison within group were performed using repeated measures ANOVA. Skewed distribution data are presented using median (M) and interquartile range (IQR), and the comparison between groups were performed using rank sum test. Comparison of count data between groups were performed using the chi-square test. *P* < 0.05 was considered a statistically significant difference.

## Results

A total of 63 patients were enrolled in this study and were randomly divided into the PCIA(*n* = 31) group and CQLB(*n* = 32) groups. One was lost to follow-up in PCIA group, one block failure and one catheter occlusion in CQLB was eliminate. At last, 30 patients in each group completed the study The Consolidated Standards of Reporting Trials (CONSORT) diagram is shown in Fig. 1. Differences in gender composition, age, body mass index (BMI), ASA classification, rate of bile duct exploration, and comorbidity between the two groups showed no significant differences(*P* > 0.05) (Table 1).

**Table 1:** Comparison of the general conditions of the two groups of patients

		CQLB group	PCIA group	*P*-value
Sex ratio [female/male, %]		13/17	13/17	> 0.99
Age (year)		50.7 ± 6.8	49.3 ± 9.1	0.62
Body Mass Index(kg/m^2^)		23.1 ± 2.7	22.4 ± 2.6	0.37
ASA classification ratio (I/II/III)		14/13/3	16/12/2	0.83
Rate of bile duct exploration [case (%)]		10 (33.3%)	10 (33.3%)	> 0.99
Comorbidity [case (%)]	Hypertension	3 (10%)	2 (6.7%)	0.86
	Diabetes	2 (6.7%)	2 (6.7%)	
	Liver cirrhosis	5 (16.7%)	6 (20%)	

No significant difference in the duration of surgery was observed between the two groups (*P* > 0.05). The consumption of propofol and remifentanil in CQLB group were significantly lower than those in PCIA group (*P* < 0.05). The time for recovery from anesthesia in CQLB group was significantly shorter than in PCIA group (*P* < 0.05) (Table 2).

**Table 2:** Comparison of the intraoperative conditions of the two groups of patients (mean ± standard deviation)

	CQLB group	PCIA group	*P*-value
Operation time (min)	202.6 ± 65.9	186.8 ± 42.7	0.65
Propofol dosage (mg·kg^−1^·h^− 1^)	4.3 ± 0.9	5.1 ± 1.1	< 0.01
Remifentanil dosage ((ug·kg^− 1^·h^− 1^)	5.1 ± 1.3	6.7 ± 1.4	< 0.01
Time for recovery from anesthesia(min)	21.0 ± 14.3	27.7 ± 19.4	0.03

As shown in Table 3 and Table 4, SBP, DBP, and HR of PCIA group at T2 were significantly higher than those in CQLB group (*P* < 0.05); no significant differences were found between the two groups at other time points (*P* > 0.05). SBP, DBP and HR were significantly higher at T2 than T1 in PCIA group (*P* < 0.05); while, only SBP and DBP were significantly higher at T2 than T1 in CQLB group (*P* < 0.05).

**Table 3:** Comparison of intraoperative SBP, DBP, and HR (mean ± standard deviation)

		T0	T1	T2
SBP (mmHg)	CQLB	133.6 ± 14.6	114.0 ± 8.8	121.8 ± 11.3^ab^
	PCIA	128.6 ± 14.3	110.7 ± 12.7	130.1 ± 13.9^b^
DBP (mmHg)	CQLB	78.3 ± 6.0	66.8 ± 6.1	70.5 ± 8.6^ab^
	PCIA	77.5 ± 7.7	67.5 ± 7.7	77.2 ± 8.0^b^
HR (bpm)	CQLB	78.4 ± 12.0	62.0 ± 9.9	62.8 ± 10.1^a^
	PCIA	78.3 ± 11.4	60.3 ± 9.1	67.1 ± 9.5^b^

Compared with PCIA group, ^a^*P* < 0.05; compared with T1, ^b^*P* < 0.05

**Table 4:** Comparison of postoperative SBP, DBP, and HR (mean ± standard deviation)

		T3	T4	T5	T6	T7	T8
SBP (mmHg)	CQLB	132.7 ± 13.7	125.8 ± 12.9	128.2 ± 14.1	121.7 ± 14.6	127.1 ± 17.5	123.3 ± 13.0
	PCIA	134.4 ± 13.7	127.8 ± 13.7	127.7 ± 17.0	115.8 ± 15.5	121.1 ± 18.3	122.4 ± 16.2
DBP (mmHg)	CQLB	69.1 ± 8.4	75.7 ± 7.4	78.3 ± 6.7	76.1 ± 9.4	77.0 ± 10.1	77.4 ± 8.2
	PCIA	69.9 ± 9.6	76.5 ± 10.6	77.3 ± 11.0	72.7 ± 11.3	74.6 ± 14.1	74.6 ± 12.1
HR (bpm)	CQLB	67.1 ± 7.7	73.3 ± 11.3	75.4 ± 10.2	78.9 ± 15.6	78.3 ± 14.4	80.2 ± 11.9
	PCIA	67.6 ± 10.4	73.1 ± 11.2	75.8 ± 10.3	79.5 ± 15.7	78.5 ± 14.2	79.8 ± 11.9

No significant differences in SBP, DBP, and HR were found between the two groups of patients at different postoperative time points

Postoperative NRS pain scores during coughing in CQLB group at all time points were significantly lower than those in PCIA group (*P* < 0.05). Postoperative NRS pain score at rest in CQLB group were generally lower than those in PCIA group at all time points, but only showed significant different at T8 time point (*P* < 0.05). Ramsay sedation scale of CQLB group at T6 time point was significant lower compared with PCIA group (*P* < 0.05), no significant different were observed at other time points (Table 5).

**Table 5:** Comparison of postoperative pain scores and sedation scale [Score, M (IQM)]

		T3	T4	T5	T6	T7	T8
NRS pain score at rest	CQLB	2.0 (2,4)	3.0 (2,4)	3.0 (2,4)	2.0 (2,3)	2.0 (1,3)	1.0 (1,2)^a^
	PCIA	3.0 (3,4)	3.0 (3,4)	3.0 (3,4)	3.0 (2,3)	3.0 (2,3)	2.0 (2,3)
NRS pain score on coughing	CQLB	4.0 (3,6)^a,^	5.0 (4,6) ^a^	5.0 (4,5) ^a^	4.0 (4,5) ^a^	3.0 (3,5) ^a^	3.0 (1,3) ^a^
	PCIA	5.0 (4,6)	5.0 (5,6)	5.0 (5,6)	5.0 (3,5)	4.0 (4,5)	4.0 (3,4)
Ramsay Sedation scale	CQLB	3.0 (2,3)	3.0 (2,3)	2.0 (2,3)^a^	2.0 (2,3)	2.0 (2,2)	2.0 (2,2)
	PCIA	3.0 (2,3)	3.0 (2,3)	3.0 (2,3)	2.0 (2,3)	2.0 (2,2)	2.0 (2,2)

Compared with the PCIA group, ^a^*P* < 0.05

The time to first flatus and first out-of-bed activity after surgery in CQLB group were significantly earlier than those in PCIA group (*P* < 0.05). No significant differences in the postoperative self-administered analgesic dose, rate of postoperative supplemental analgesic, or incidences of analgesic-related adverse effects were found between the two groups (*P* > 0.05) (Table 6).

**Table 6:** Comparison of postoperative conditions between the two groups of patients

	CQLB group	PCIA group	*P*-value
Time to first out-of-bed activity after surgery (h)	73.2 ± 24.9	85.7 ± 23.0	0.03
Time to first flatus after surgery (h)	61.7 ± 18.1	70.1 ± 15	0.03
Self-administered analgesic counts [M (IQM)]	3 (1, 4)	3 (2, 4)	0.38
Rate of supplemental analgesic use [case (%)]	4 (13.3%)	6 (20%)	0.73

The incidence of postoperative advers effects such as poseoperative agitation,respiratory depression,nausea, vomiting and dizziness were compared separately between the two groups ,no significant were founded (*P* > 0.05) (Table 7).

**Table 7:** Comparison of empathic rate of analgesic-related adverse effects between the two groups of patients [case (%)]

	CQLB group	PCIA group	*P*-value
Postoperative agitation	0 (0%)	1 (3.3%)	> 0.99
Respiratory depression	0 (0%)	1 (3.3%)	> 0.99
Nausea	4 (13.3%)	7 (23.3%)	0.51
Vomiting	2 (6.6%)	4 (13.3%)	0.67
Dizziness	0 (0%)	3 (10%)	0.24

## Discussion

In this study, we found CQLB was a safe and effective regimen of postoperative analgesia for open live resection. The patients in CQLB group received adequate analgesia and recovered earlier than the patients in PCIA group.

QL block is an emerging nerve block technique developed from transversalis fascia plane block. Compared with transversalis fascia plane block, the QL block site is closer to the spine, with significantly wider block range, better analgesic effect, and longer analgesic duration [15]. In this study, all patients, except one, underwent QL block successfully, and the right abdominal pain relief range completely covered the area innervated by T7–T12 thoracic nerves. The possibility of success was 96.9% (31/32). No intrathecal block, local anesthetic toxicity, or weakened lower right limb muscle strength were observed before anesthesia induction and during postoperative follow-up. The block range of QL block can be affected by various factors, such as puncture level, block approach, and drug volume [16–18]. Thus, different block planes and approaches can be selected according to the needs for analgesia. Open liver resection usually involves an arc-shaped incision, which is parallel to the right rib margin and 2 cm below the xiphoid process. The outside of the incision often extends to the anterior axillary line. Transection of the anterior branches of multiple spinal nerves caused by surgical incision and traction injury of intercostal nerves caused by prolonged traction of the ribs can cause intensive postoperative pain and are the major sources of postoperative pain. To adequately block the pain transmission of the incision, here we chose to perform anterior QL block at L2 transverse processes. Studies have reported several advantages of this approach. For example, (1) local anesthetics during anterior QL block can spread to the thoracic fascia to create a higher block plane, and it is believed that anterior QL block can alleviate visceral pain [13, 19]. (2) Compared with the commonly used puncture level under the L3 transverse processes, an approach at a higher level can achieve a wider range of block towards cephalad while avoiding the spread of anesthetics to the lumbar plexus, causing weakness of the lower extremities [20]. (3) Unlike the subcostal QL block, during which the tip of the needle is very close to the kidney, it seems much safer to perform continuous quadratus lumborum block in the L2 transverse process level because the quadratus lumborum is thick enough to protect the kidney.

Our results showed that the hemodynamic fluctuations during skin incision in CQLB group were significantly less pronounced than that in PCIA group (Table 3). In addition, the intraoperative propofol and remifentanil consumption of CQLB group were significantly lower (Table 2), and the time for the recovery from anesthesia in CQLB group was significantly earlier than PCIA group (Table 2). These results were consistent with the findings in the study of Baidya et al. [21], suggesting that anterior QL block combined with general anesthesia alleviated perioperative stress response and reduced the required dose of general anesthetic drugs, which promoted the patient’s recovery from anesthesia.

Pain scores at rest of two groups in this study were significantly different only at 48 h after surgery (Table 5). However, pain scores upon coughing in the CQLB group at different postoperative time points were significantly lower than those in the PCIA group (Table 5). This can be explained by the nerves involved in pain transmission in the lateral abdominal wall of the surgical site were blocked, and the painful stimuli due to traction of the wound during coughing could not be efficiently transferred to the central nervous system. In PCIA group, the median number of pain score on coughing was 5 in the first day of post-operation (Table 5), which suggested that about half patients may not receive adequate analgesia in the early postoperative period. Since the dose of sufentanil in this group is 0.05 μg·kg^− 1^·h^− 1^, which was considered high in most clinical practices [22, 23], increasing the dose of sufentanil may raise risk of side effects.

Results (Table 6) showed that patients in CQLB group had shorter time to first anal flatus than patients in PCIA group, which mean that patients in CQLB group could be allowed to oral intake much earlier. The longer time to first flatus may be one side effects of the opioids, which are associated with constipation and slowing bowel movement. Additionally, the patients with PCIA need more time to commence their first out-of-bed activity. Early ambulation and oral intake are keys to achieve the enhanced recovery after surgery. The enhanced recovery after surgery (ERAS) has become a popular management mode for contemporary surgical procedures, which aims to reduce perioperative complications, accelerate postoperative recovery, and shorten the hospital stays of patients [24]. A reasonable perioperative analgesia program can effectively control the perioperative stress response, reduce postoperative pain, enable the restoration of oral feeding and out-of-bed activities, and promote patient’s rehabilitation, so it is an important part of ERAS. Heavy consumption of opioids has been associated with detrimental effects to the patients, such as respiratory embarrassment, opioid associated ileus and addiction, etc. Thus, it has become a clinical consensus in favor of use of opioid alternatives for analgesia. Peripheral nerve block techniques have obvious advantages in reducing perioperative stress response and alleviating pain during early ambulation. It has fewer adverse effects and did not affect gastrointestinal motility. ERAS guidelines for many disciplines have recommended the regional block techniques as adjuvant analgesia during the perioperative period [8, 25], which can significantly reduce the use of opioid analgesics. Appropriate regional block techniques can completely replace opioids. In this study, patients in the CQLB group required fewer general anesthetics, achieved better overall analgesic effect after surgery, and had significantly shortened time to first out-of-bed activity and time to first flatus after surgery. The improved postoperative analgesic effect may promote early ambulation of the patients, and shorter time to flatus means that the patient may recommence eating and swallowing sooner. These results confirmed our hypothesis that CQLB is a hopeful perioperative analgesic method, promoting rapid recovery of the patients after open abdominal liver surgery.

Although it has been clearly established that epidural analgesia is able to provide perfect analgesic effect, this technique still has several potential flaws, such as perioperative hypotension, which means vasopressors are potentially needed and the possibility of acute kidney failure are increasing [26]. Neurological complications, which include epidural hematoma and abscess with an incidence of one in 1000–6000 for thoracic epidurals [27–29], may cause serious consequences. Unlike the epidural anesthesia, local anesthetic of QL block does not enter the spinal column but still unilaterally blocks the spinal nerves from T6–T9 to L1-L3 [30], thus QL block may offer greater safety and adequate analgesia. In addition, the success rates of CQLB exceeded 96.9% (31/32) with ultrasound guidance, while the epidural inadequate analgesia rates may be up to 30% [31].

Nevertheless, this study also has some limitations. With reference to the previous literature, this study only used 0.6 ml·kg^− 1^0.4% ropivacaine hydrochloride in preoperative nerve block and postoperative continuous infusion of 5 ml·h^− 1^ 0.2% ropivacaine hydrochloride [18, 21, 32], without any observable local anesthetic toxicity. However, since QL block is still in the exploratory stage, there is no consensus regarding optimal drug concentration and volume, and further verification of optimal drug volumes of this study will be necessary. QL block has a slow onset of action. A study by Johnston et al. has shown that the nerve block effect only occurs 21 min after blockade [12]. However, the time to achieve the maximum and stable block range is still unclear. Due to safety and time limitations, this study was designed to evaluate the nerve block plane 30 min after the block, but whether the block range had been stabilized at this time will need further study. Due to the small sample size of this study, for adverse effects we only observed postoperative nausea and vomiting, and no significant differences were found between the two groups, probably because of the small sample size.

## Conclusion

In conclusion, perioperative use of ultrasound-guided continuous anterior QL in the patient undergoing open liver surgery significantly reduced the intraoperative dose of anesthetics in general anesthesia, alleviated postoperative pain during coughing, shortened the time to first flatus and time to first out-of-bed activity after surgery, and promoted rapid postoperative recovery of the patient.

## Abbreviations

ANOVA: Analysis of variance
ASA: America Society of Anesthesiologist
BMI: Body Mass Index
CQLB: Continuous anterior quadratus lumborum block
DBP: Diastolic Blood Pressure
HR: Heart Rate
L1, L3: First lumbar vertebra, Third lumbar vertebra
NRS: Numerical Rating Scale
NYHA: New York Heart Association
PCIA: Patient-controlled intravenous analgesia
QL: Quadratus lumborum
SBP: Systolic Blood Pressure
T6, T9: Sixth thoracic vertebra, Ninth thoracic vertebra
